# The impact of the COVID-19 pandemic on the prevalence and risk factors of workplace violence among healthcare workers in China

**DOI:** 10.3389/fpubh.2022.938423

**Published:** 2022-07-25

**Authors:** Miao Qi, Xiuli Hu, Jun Liu, Jing Wen, Xue Hu, Zhizhong Wang, Xiuquan Shi

**Affiliations:** ^1^Department of Epidemiology and Health Statistics, School of Public Health, Zunyi Medical University, Zunyi, China; ^2^Department of Preventive Medicine, School of Public Health, Zunyi Medical University, Zunyi, China; ^3^Department of Epidemiology and Health Statistics, School of Public Health and Management, Ningxia Medical University, Yinchuan, China; ^4^Department of Epidemiology and Health Statistics, School of Public Health, Guangdong Medical University, Dongguan, China; ^5^Center for Injury Research and Policy & Center for Pediatric Trauma Research, The Research Institute at Nationwide Children's Hospital, The Ohio State University College of Medicine, Columbus, OH, United States

**Keywords:** COVID-19, workplace violence, healthcare workers, prevalence, risk factors, China

## Abstract

**Background:**

The pandemic of COVID-19 has significantly increased the burden on healthcare workers and potentially affect their risk of workplace violence (WPV). This study aimed to explore the prevalence and risk factors of WPV among healthcare workers during the peaking and the remission of the COVID-19 pandemic in China.

**Methods:**

Using the snowball method, a repeated online questionnaire survey was conducted among Chinese healthcare workers from March 27th to April 26th in 2020 and 2021, respectively. Data included healthcare workers' socio-demographic and occupational characteristics, psychological status, and workplace violence.

**Results:**

A total of 3006 samples in 2020 and 3465 samples in 2021 were analyzed. In 2020, the prevalence of WPV and witnessing colleagues suffering from WPV among healthcare workers were 64.2% and 79.7% respectively. Compared with 2020, the prevalence decreased by 11.0% and 14.4% in 2021, respectively. Logistic regression showed that WPV in 2020 was influenced by males, long working experience, working in the psychiatric department, direct contact with COVID-19 patients, self-discovery of medical errors, moral injury, depression, and anxiety (minimum OR = 1.22, maximum OR = 2.82). While risk factors of WPV in 2021 included males, working in psychiatric departments, self-discovery of medical errors, moral injury, depression, and anxiety (minimum OR = 1.33, maximum OR = 3.32); and protective factors were holding a master's degree (OR = 0.78) and working in other departments (OR = 0.54).

**Conclusion:**

This study retains the common effects of WPV among healthcare workers, though after the baptism of the COVID-19 pandemic, the prevalence of WPV among healthcare workers decreased; however, part of the influencing factors changed. In addition, COVID-19 has seriously affected the mental health of healthcare workers, and the effect of mental health problems on WPV should also attract more attention.

## Introduction

Workplace violence (WPV) refers to any destructive behaviors toward employees' safety, happiness, and health in the workplace, including physical attacks, threats, intimidation, emotional abuse, verbal sexual harassment, and sexual harassment, ranging from threats and insults to personal attacks and even killings (1–3). This phenomenon can be seen as the result of social intricacy and emotional guidance. As a working environment with high mobility, special emotional ups and downs, and complex social components, hospitals are high-risk places for WPV globally (4, 5).

In 2019, the Occupational Safety and Health Administration (OSHA) reported that the prevalence of WPV in the healthcare environment was twice that of the private sector (6). WPV in hospitals is associated with several negative consequences, including worsened physical and mental health (7–9), decreased job satisfaction and quality of life (10–12), and increased burnout and turnover intention (12, 13), which in turn threaten the quality of health care and the patients' safety (14–16).

The pandemic of COVID-19 is undoubtedly a huge challenge for the health care system. To maintain global public health safety, countless front-line healthcare workers enrolled in exhaustion fighting against the pandemic (17). Due to the unprecedented overload and different levels of pressure, the risk of infection, moral injury, depression, anxiety, and WPV among healthcare workers increases (18–20). However, when the healthcare workers are exhausted, they can not provide the best medical care and are more likely to make mistakes, which should further increase the risk of conflict between healthcare workers and patients (21). The serious consequences caused by WPV on healthcare workers inevitably increase the burden on the medical and health system, which is not conducive to jointly fighting the epidemic situation and maintaining the health and safety of all humankind. The World Health Organization (WHO) also put forward “the safety of health workers: it is more important than ever before” on September 17th, 2020, on the occasion of World Patient Safety Day, indicating that the health, safety, and wellbeing of health workers are prerequisites for effectively coping with COVID-19 pandemic and other public health emergencies, and providing basic health services (22).

Today, COVID-19 is still wreaking havoc in every corner throughout the world, and the topic of the epidemic has been firmly in the headlines of the world. As a big country with a population of 1.4 billion, the WPV faced by healthcare workers in China has an unstable development trend, and WPV related factors are complicated and diverse.

In this study, we aim to explore whether the prevalence of WPV among healthcare workers and related factors have changed pleasantly after the baptism of the COVID-19 epidemic in the past 2 years and to provide evidence for formulating preventive measures. We assume that through the experience of fighting the pandemic of the COVID-19 together and the positive publicity of the media to medical workers, the prevalence of WPV among medical workers has decreased correspondingly.

## Materials and methods

### Research design

In order to comply with and support the prevention and control of the epidemic situation in COVID-19, repeat online survey was conducted among two hospital-based samples enrolled by snowball convenient sampling in March 2020 and March 2021. More complete details on participant recruitment have been described elsewhere (23). Participants need to meet the following criteria to be eligible for inclusion in the study, (1) Doctors or nurses who have worked in hospitals in mainland China for at least 2 years; (2) Have the corresponding practicing qualification certificate; (3) Be able to use the Internet normally and complete online questionnaires; (4) Select the consent option of the online report (I agree to participate in the research). Exclude those who have left their jobs for half a year or over for any reason(s) in the past 2 years. The research was approved by the Institutional Review Committee of Ningxia Medical University (No.2020-112). All information investigations are anonymous and confidential.

Invitations and online questionnaires are created using the “Questionnaires” online survey platform, which is opened by nearly one billion users every day and sent by WeChat, the most popular instant messaging software in China (24). In this stage, we carry out quality control: (1) When a participant completes the questionnaire in <250 secs, the questionnaire will be marked as invalid; (2) The smart device client can fill in the questionnaire only once. During the survey in 2020, a total of 4,003 people responded to the invitation to participate in the recruitment, of which 28 participants did not agree to participate in the study and got 3,975 online questionnaires. In the process of data cleaning, 968 samples were excluded, which were submitted repeatedly, worked for <2 years, scored the same or similar in all projects, and had more than two missing items. Finally, 3006 samples were included in the analysis. Similar to the above method steps, in 2021, a total of 4,025 people responded to the invitation to participate in the recruitment, of which 8 participants did not agree to participate in the study, and 552 data were cleared out, and finally 3,465 samples were included in the analysis.

### Measures

The data of socio-demographic and occupational characteristics were collected, including gender, age, education level, marital status, professional field, work field, working years, contact with COVID-19 patients, and so on.

WPV is measured by asking two yes or no questions: (1) Have you ever been physically or verbally attacked by your patients or their close relatives? (2) Have you witnessed your colleagues being physically or verbally attacked by patients or their close relatives? It is important to note that two yes or no questions were added in 2021. (3) Have you been attacked by your patients or their relatives in the past year? (4) In the past year, did you know that your colleagues were attacked by patients or their relatives?

Moral Injury Symptoms Scale–Health Professional version (MISS-HP) (25): ten-item instrument covering 10 dimensions, including betrayal, guilt, shame, moral concerns, loss of trust, loss of meaning, difficulty forgiving, self-condemnation, faith struggle, and loss of faith. Respond to ten options from 1 to 10, indicating agreement or disagreement. The total score is from 10 to 100, and the higher score indicates more MI symptoms (26). MISS-HP is translated into Chinese according to the standard procedure (27). At present, the Cronbach's value in the sample is 0.71 for nurses and 0.70 for doctors, and the internal consistency coefficient of the retest is 0.77, thus, the reliability and validity of this scale are acceptable (23).

The 9-item Patient Health Questionnaire (PHQ-9): there are 9 items to evaluate and monitor the severity of depression, and the frequency of each symptom from 0 (no at all) to 3 (almost every day) in the past 2 weeks is scored on a 4-point scale. The total scores are as follows: lowest/no depression (0–4), mild depression (5–9), moderate depression (10–14), moderate-severe depression (15–19) or severe depression (20–28). According to the total score, we classify depression into dichotomous variables by <10 (no or mild depression) of the PHQ-9 total score. The Cronbach's alpha in the present sample was 0.91 for PHQ-9. The Chinese version of the PHQ-9 scale has strong internal reliability and retest reliability, as well as structural validity and factor structural validity in patients and general population (29).

The 7-item Generalized Anxiety Disorder (GAD-7): there are 7 items to measure the severity of generalized anxiety disorder. According to the frequency of each symptom in the past 2 weeks from 0 (no symptom at all) to 3 (almost every day), score each item on a 4-point scale. Elevated scores are classified as mild anxiety (5–9), moderate anxiety (10–14), and severe anxiety (15–21, 30). When the score is <10, we decide that there is no anxiety. The Cronbach's alpha for the present sample was 0.94. The Chinese version of the GAD-7 scale is highly effective and reliable in medical patients and the general population (31).

### Statistical analyses

The socio-demographic characteristics, clinical work-related characteristics, and psychological state are reported in numbers and percentages. Chi-square test was used to compare the socio-demographic characteristics, clinical work-related characteristics, and psychological states between groups who suffered from WPV and those who did not, and those who witnessed and did not witness WPV. Multivariate logistic regression was used to analyze the influencing factors of personal experience or witness of WPV from independent demographic characteristics, clinical variables, and psychological state. The IBM SPSS (Version24.0; IBM Corporation, Armonk, NY, USA) was used to perform all analyses. Drawing forest map with GraphPad Prism (Version 8.3; GraphPad Software Inc., Motulsky HJ, San Diego, CA, USA). *P*-value of < 0.05 was considered statistically significant (two-tailed).

## Results

### Socio-demographic, occupational characteristics, and psychological state of the study participants

Table 1 shows the socio-demographic, occupational characteristics, psychological state of participants, and the distribution of the prevalence of WPV in 2020 and 2021. In 2020, there were 3,006 samples, of which 1,049 (34.9%) were male, mostly 31–40 years old (40.1%), married (75.4%), with a bachelor's degree (67.5%), and more than 10 years of work experience (51.3%). A total of 1,931 (64.2%) reported having personally experienced WPV, of whom 475 (15.8%) said they had direct contact with a COVID-19 patient, 1,140 (37.9%) said they had made a medical error, and 1,149 (38.2%) said they had suffered moral harm, 1,159 (38.6%) reported moderate or severe depressive symptoms, and 624 (20.8%) reported moderate or severe anxiety symptoms. In addition, 2397 (79.7%) witnessed colleagues being subjected to WPV. In 2021, there were 3,465 samples, with a similar distribution of demographic characteristics as in 2020. Of the 1,844 (53.2%) who reported experiencing, 1,500 (81.3%) reported moderate or severe depressive symptoms. Meanwhile, 2,263 (65.3%) witnessed colleagues being subjected to WPV. It is worth noting that in 2021, 30.2% of medical personnel reported having been subjected to WPV in the past year.

**Table 1:** Socio-demographic, occupational characteristics of participants, and prevalence of workplace violence in year 2020 and 2021 [*n* (%)].

**Variables**	**2020 (***N*_1_ = **3,006)**			**2021 (***N*_2_ = **3,465)**		
	**Overall**	**Violence (Yes)**	**Witness (Yes)**	**Overall**	**Violence (Yes)**	**Witness (Yes)**
Total	3,006 (100.0)	1,931 (64.2)	2,397 (79.7)	3,465 (100.0)	1,844 (53.2)	2,263 (65.3)
Gender, males	1,049 (34.9)	761 (39.4)	891 (37.2)	853 (24.6)	514 (27.9)	628 (27.8)
Age (years)						
≤ 30	1,085 (36.1)	602 (31.2)	792 (33.0)	1,108 (32.0)	487 (26.4)	622 (27.5)
31–40	1,206 (40.1)	810 (41.9)	975 (40.7)	1,501 (43.3)	819 (44.4)	982 (43.4)
41–50	572 (19.0)	412 (21.3)	508 (21.2)	623 (18.0)	385 (20.9)	476 (21.0)
>50	143 (4.8)	107 (5.5)	122 (5.1)	233 (6.7)	153 (8.3)	183 (8.1)
Education						
Bachelor	2,029 (67.5)	1,296 (67.1)	1,590 (66.3)	2,812 (81.2)	1,500 (81.3)	1,830 (80.9)
Master	813 (27.0)	521 (27.0)	664 (27.7)	541 (15.6)	276 (15.0)	360 (15.9)
Ph.D	164 (5.5)	114 (5.9)	143 (6.0)	112 (3.2)	68 (3.7)	73 (3.2)
Marital status						
Unmarried	656 (21.8)	365 (18.9)	476 (19.9)	734 (21.2)	345 (18.7)	442 (19.5)
Married	2,266 (75.4)	1,506 (78.0)	1,849 (77.1)	2,614 (75.4)	1,428 (77.4)	1,739 (76.8)
Divorced/widow	84 (2.8)	60 (3.1)	72 (3.0)	117 (3.4)	71 (3.9)	82 (3.6)
Specialty						
Nurse	540 (18.0)	314 (16.3)	416 (17.4)	509 (14.7)	223 (12.1)	312 (13.8)
Internal medicine	583 (19.4)	337 (17.5)	422 (17.6)	1,549 (44.7)	828 (44.9)	991 (43.8)
Obstetrics/gynecology/pediatrics	1,043 (34.7)	691 (35.8)	857 (35.8)	731 (21.1)	381 (20.7)	496 (21.9)
Surgery	290 (9.6)	189 (9.8)	229 (9.6)	170 (4.9)	93 (5.0)	109 (4.8)
Psychiatry	343 (11.4)	245 (12.7)	295 (12.3)	294 (8.5)	195 (10.6)	217 (9.6)
Other	207 (6.9)	155 (8.0)	178 (7.4)	212 (6.1)	124 (6.7)	138 (6.1)
Length in practice (years)						
≤ 5	969 (32.2)	527 (27.3)	708 (29.5)	827 (23.9)	358 (19.4)	467 (20.6)
6–9	495 (16.5)	314 (16.3)	390 (16.3)	608 (17.5)	299 (16.2)	357 (15.8)
≥10	1,542 (51.3)	1,090 (56.4)	1,299 (54.2)	2,030 (58.6)	1,187 (64.4)	1,439 (63.6)
COVID−19 patient care, yes	668 (22.2)	475 (24.6)	559 (23.3)	462 (13.3)	262 (14.2)	309 (13.7)
Medical error (self–discovery), yes	1,495 (49.7)	1,140 (59.0)	1,305 (54.4)	1,339 (38.6)	918 (49.8)	1,043 (46.1)
Moral injury, yes	1,695 (56.4)	1,149 (59.5)	1,390 (58.0)	1,270 (36.7)	756 (41.0)	873 (38.6)
Depression, yes	1,663 (55.3)	1,159 (60.0)	1,384 (57.7)	2,621 (75.6)	1,500 (81.3)	1,820 (80.4)
Anxiety, yes	860 (28.6)	624 (32.3)	724 (30.2)	712 (20.5)	447 (24.2)	532 (23.5)

*Violence-Participants had been physically or verbally attacked by patients or their close relatives; Witness-Participants witnessed co-workers being physically or verbally attacked by patients or their close relatives*.

### Multiple logistic regression analysis of experiencing WPV and witnessing colleagues' WPV

**Figure 1:**
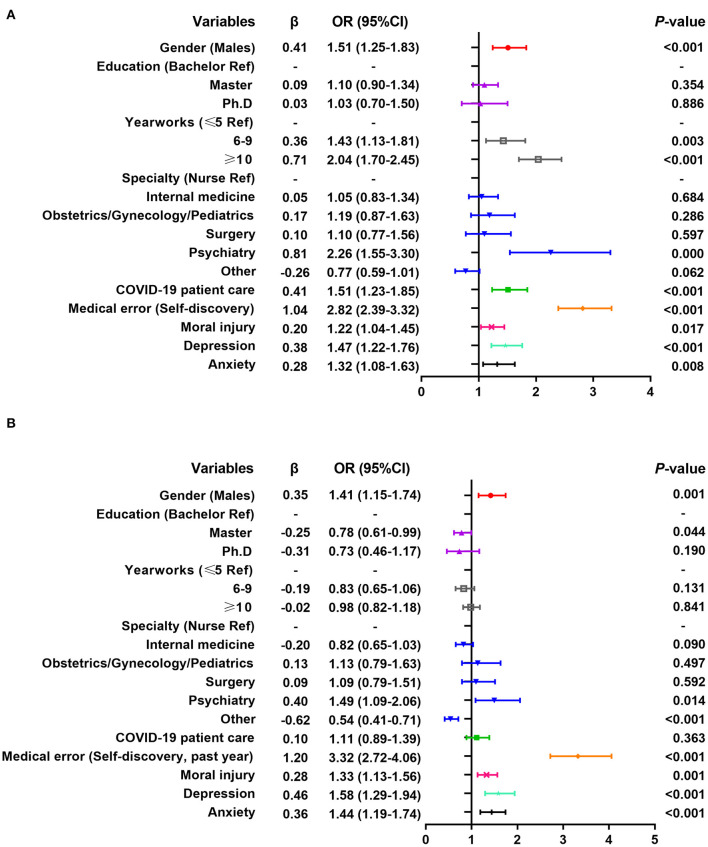
Multiple logistic regression analysis of related factors of workplace violence experienced by healthcare workers. **(A)** Results of the 2020 survey; **(B)** Results of the 2021 survey.

**Figure 2:**
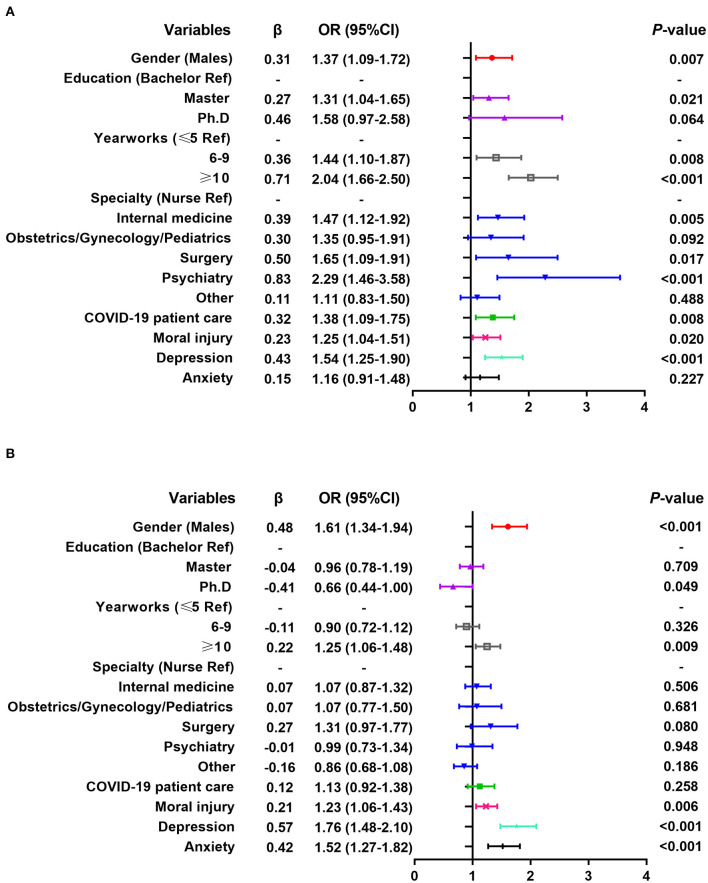
Multiple logistic regression analysis of related factors of healthcare workers witnessing colleagues' workplace violence. **(A)** Results of the 2020 survey; **(B)** Results of the 2021 survey.

Multivariate logistic regression analysis shows that the personal experience of WPV surveyed in 2020 is positively correlated with male, long working experience (6–9, ≥10 years), working in the psychiatric department, direct contact with COVID-19 patients, self-discovery of medical errors, moral injury, depression, and anxiety (the detailed odds ratios [OR] and *P*-values see Figure 1A). Witnessing colleagues' WPV is positively correlated with male, master's degree, long working experience (6–9, ≥10 years), working in internal medicine, surgery, and psychiatry departments, direct contact with COVID-19 patients, moral injury and depression (see Figure 2A).

Multivariate logistic regression analysis shows that the personal experience of WPV surveyed in 2021 is positively correlated with males, working in psychiatric departments, self-discovery of medical errors in the past year, moral injury, depression and anxiety, and negatively correlated with master's degree and working in other departments (see Figure 1B). Witnessing colleagues' WPV is positively correlated with males, long working experience (≥10 years), moral injury, depression, and anxiety (see Figure 2B).

## Discussion

WPV among healthcare workers has become a serious global problem and challenge, which is also the main occupational hazard faced by healthcare workers around the world. On a global scale, verbal and physical violence against healthcare workers has reached a high level. Violence against healthcare workers has become an international emergency, destroying the foundation of the health system and seriously affecting the health of patients (32). In our study, the incidence of WPV experienced and witnessed by healthcare workers, which was in the range from 50.0 to 84.2% reported in the past (33, 34), similar to the prevalence of other outcomes during the COVID-19 pandemic, such as the combined prevalence of WPV among healthcare workers in Asia and the America at 44.0 and 58.0%, respectively (35). But lacking the comparable studies a year or more after the peak of COVID-19. It is worth exploring that the reasons for the high incidence of WPV in 2020 may be the fear of patients and their families about COVID-19, the anxiety and lack of understanding about this serious respiratory infectious disease may potentially increase the number of WPV cases (36). All of these negative emotions are vented on the health staff who have the most contact with the sick, andthe collapse of the health system and the heavy workload of health workers might also increase the risk of WPV (37).

The causes of WPV against healthcare workers are very complex. Consistent with many previous research results (14, 38, 39), in this survey, male medical workers are more likely to experience WPV than their female counterparts. Male clinicians are more likely to suffer from physical violence than female clinicians, which may be related to male healthcare workers describing events more often than female healthcare workers, including witnessing colleagues' experiences (38). Moreover, clinicians of different genders are different in personality and communication with patients. Female clinicians are often softer in language, and more polite and sympathetic (40).The results of the personal experience of WPV surveyed in 2020 are consistent with those of earlier studies, and there is no connection between education and WPV (41), but in 2021, having a master's degree should become a protective factor. In witnessing colleagues being subjected to WPV, having a master's degree in 2020 is a risk factor, while only having a doctor's degree in 2021 is a protective factor. It may be that healthcare workers have received more education and professional training, and can better cope with the diseases and emotions of patients under the situation of COVID-19.

However, it should be noted that in the 2020 survey, healthcare workers with long working experience are more likely to encounter WPV, which is consistent with previous studies (42). Compared with junior staff, more experienced healthcare workers may have access to more difficult and challenging patients, and the aggravation of workload and difficulties may affect their health and service quality, which increases the possibility of violence (39). In addition, research shows that patients often have higher expectations for experienced healthcare workers (43), and unmet expectations constitute the main risk of attack (44). In the 2021 survey, the personal experience of WPV was not related to the work experience of healthcare workers. It may be due to the sudden COVID-19 outbreak that all healthcare workers, especially junior staff, are growing rapidly. The joint efforts of personnel at all levels have been appreciated and respected by patients. Compared with other majors, the prevalence of WPV in psychiatry in this study is higher, which is consistent with previous findings (45). Because of the special nature of patients, patients in psychiatric departments usually suffer from worsening symptoms during treatment, and they are more likely to use violence and try to hurt psychiatrists (33). In addition, inappropriate mental health service resources aggravate conflicts and violence (46).

At first, caring for COVID-19 patients was highly correlated with WPV. In the early stage of the COVID-19 pandemic, due to the public's excessive fear and worry about COVID-19 and the influence of a large number of false news, many front-line healthcare workers were mistakenly regarded as disseminators of the virus, and were verbally or even physically abused by the public (18, 47–49). In the 2021 survey, taking care of patients infected with COVID-19 is no longer a risk factor for WPV, whether personal experience or witnessing colleagues' WPV. We suspect that this has something to do with the public's clear understanding of COVID-19, the great efforts of healthcare workers in fighting the COVID-19 pandemic, and the positive publicity of the state, government, and media. In addition, our research also found that there were differences in influencing factors between health workers who experienced WPV and those who witnessed WPV. It may be that both the psychological and physical injuries suffered by one's own experience are the most direct and serious, while witnessing WPV is mainly psychological but not physical injuries, so some influencing factors are different.

Notably, this study found that self-reported medical errors, moral injury, depression, and anxiety were positively correlated with personal experience or witnessing WPV at two surveyed time points. Studies have found that healthcare workers under high-intensity work and under high-level psychological pressure are more likely to make medical errors and have poor interpersonal communication with patients and their families, which will make them face a high risk of WPV (50). During the COVID-19 pandemic, the prevalence of moral injury, depression, and anxiety among healthcare workers were higher than before, because they not only faced high-intensity and difficult work pressure but also were more likely to be infected with COVID-19, fearing that the virus would spread to their relatives and colleagues, which would seriously affect their mental health (37). Our results show that the prevalence of depression (55.3%) among healthcare workers in China during the COVID-19 pandemic was higher than in some other meta-analyses and epidemiological surveys during the pandemic. For example, it was much higher than the comprehensive prevalence of depression among front-line medical staff during the outbreak of COVID-19 in Spain (33.0%) (51), Southeast Asia (14.0%) (52), and a separate meta-analysis from China (32.0%) (53). Likewise, it was higher than the comprehensive prevalence of depression (21.7%) among healthcare workers in 21 countries reported in the meta-analysis report by Li et al (54). The prevalence of anxiety (28.6%) among healthcare workers in China during the COVID-19 pandemic reported in our study was close to the prevalence of anxiety (29.0%) among front-line medical staff in a separate meta-analysis in China (53). However, it was higher than the combined prevalence of anxiety (23.0%) among front-line medical staff in Southeast Asia (52) and the combined prevalence of anxiety (22.1%) among healthcare workers covering 21 countries (54). Surprisingly, it was significantly lower than the combined prevalence of anxiety among front-line medical staff in Spain (46.0%) (51), Africa (51.0%) (55) and among healthcare workers in Italy (57.0%) (56). However, we found that the trends in the prevalence of depression and anxiety among healthcare workers in China 1 year after the COVID-19 pandemic we report (55.3% to 75.6%, 28.6% to 20.5%) were consistent with those the longitudinal single-center studies of frontline emergency department healthcare workers in the hospital's COVID-19 pandemic results (25.3% to 28.6%, 30.7% to 27.0%) reported by Th'ng et al (57).

Overall, we consider that due to the different survey objects (e.g., all health personnel, front-line health personnel, general health personnel), the survey time (e.g., during the COVID-19 crisis and 1 year after the COVID-19 peak), survey methods (cross-sectional and longitudinal studies), and the evaluation tools (e.g., PHQ, SDS, GAD and SAS), the results of direct comparison need to be cautious. In addition, our study reported a higher prevalence of depression than most other studies, and had the opposite finding that depression was more common than anxiety symptoms. The reason may be related to that China has a large population, during the Spring Festival travel, the population flow is large, the virus spreads rapidly, the health system was overloaded, medical resources are scarce, and Chinese healthcare workers were faced with unknown fear and great pressure at the early stage. And reminds us that the impact of the new crown epidemic has made healthcare workers' mental health problems worse, and now more than ever, the need to improve healthcare workers has exacerbated this situation. The need to maintain and promote the mental health of healthcare workers is now more than ever.

There is insufficient research on the relationship between the mental health status of health workers and WPV during the COVID-19 outbreak, especially on the long-term impact of 1 year or more after the peak of COVID-19. Several studies have previously reported on the psychological outcomes of healthcare workers during the COVID-19 pandemic (53, 58), and some studies have reported WPV against healthcare workers during the COVID-19 pandemic (35), but studies on the relationship between the two outcomes are lacking, especially in different times of COVID-19. We hypothesized that the mental health status of health workers and WPV were interactive. On the one hand, WPV may reduce the enthusiasm and satisfaction of healthcare workers, and lead to moral injury, depression, and anxiety (13, 59). On the other hand, these negative effects may affect the work quality and results, which in turn increases the risk of WPV and further affects the safety and health of patients (14–16). Therefore, more attention and targeted multidisciplinary interventions are needed to combine addressing mental health issues with WPV, which has achieved the effect of “1+1>2”.

The merits of the present study include large sample size, wide survey area, and representative survey time. However, several limitations in this study exist. First, the participants were enrolled by snowball and there was an obvious gender difference in the samples, which would affect the extrapolation of the results. Second, when exploring the impact of the COVID-19 pandemic on WPV, other potential factors such as social support, media publicity, and public awareness were not evaluated, which may lead to overestimation or underestimation of this relationship. Third, although it is a repeated survey of 2 years, it is not a follow-up cohort. The possible mutual causation between the psychological/psychiatric variables (such as moral injury, depression, and anxiety) and WPV is still unclear, the interpretation of the findings should be cautious. Finally, WPV is self-reported through simple two-category questions and the types of WPV suffered are not classified, so it is necessary to measure the report results more objectively.

## Conclusions

In conclusion, our study reveals the common effects of WPV among healthcare workers, such as males, long working hours, working in the psychiatric department, and other factors. The results suggest that after the baptism of the COVID-19 epidemic, the prevalence of WPV among healthcare workers has been reduced. However, as a sudden, special, and serious event, whether the COVID-19 pandemic will increase or reduce the risk of WPV among healthcare workers is related to healthcare workers themselves, the control situation of the epidemic, the length of time, government policies, media publicity, and public awareness, etc., which needs more researches to verify. In addition, COVID-19 has seriously affected the mental health of healthcare workers, but it is still lacked of researches on the long-term effects. Meanwhile, the relationship between mental health problems and WPV in healthcare workers should be paid more attention to and further discussed.

## Data availability statement

The original contributions presented in the study are included in the article/supplementary material, further inquiries can be directed to the corresponding author/s.

## Ethics statement

This research was approved by the Institutional Review Committee of Ningxia Medical University (No.2020-112). The patients/participants provided their written informed consent to participate in this study.

## Author contributions

MQ, ZW, and XS: conceptualization. ZW: data curation and project administration. MQ: writing–original draft. XS and ZW: funding acquisition and writing–review & editing. MQ and XiH: formal analysis. MQ and XS: methodology. JL, JW, and XuH: visualization. All authors have read and agreed to the published version of the manuscript.

## Funding

This work was supported by the Science and Technology Support Program of Guizhou province (Project number: [2020]4Y171, Recipient: XS) and the China Medical Board (Project number: CMB16-254, Recipient: ZW).

## Conflict of interest

**T**he authors declare that the research was conducted in the absence of any commercial or financial relationships that could be construed as a potential conflict of interest.

## Publisher's note

All claims expressed in this article are solely those of the authors and do not necessarily represent those of their affiliated organizations, or those of the publisher, the editors and the reviewers. Any product that may be evaluated in this article, or claim that may be made by its manufacturer, is not guaranteed or endorsed by the publisher.
